# The effect of dietary camelina, flaxseed, and canola oil supplementation on skin fatty acid profile and immune and inflammatory responses in healthy adult horses

**DOI:** 10.1093/jas/skaf025

**Published:** 2025-02-04

**Authors:** Samantha Hartwig, Scarlett Burron, Taylor Richards, Alexandra Rankovic, David W L Ma, Wendy Pearson, Jennifer Ellis, Luciano Trevizan, Dave J Seymour, Anna K Shoveller

**Affiliations:** Department of Animal Biosciences, University of Guelph, Guelph, ON, Canada N1G 2W1; Department of Animal Biosciences, University of Guelph, Guelph, ON, Canada N1G 2W1; Department of Animal Biosciences, University of Guelph, Guelph, ON, Canada N1G 2W1; Department of Animal Biosciences, University of Guelph, Guelph, ON, Canada N1G 2W1; Department of Human Health and Nutritional Sciences, University of Guelph, Guelph, ON, Canada N1G 2W1; Department of Animal Biosciences, University of Guelph, Guelph, ON, Canada N1G 2W1; Department of Animal Biosciences, University of Guelph, Guelph, ON, Canada N1G 2W1; Departamento de Zootecnia, Universidade Federal do Rio Grande do Sul, Porto Alegre 91540-000, Rio Grande do Sul, Brazil; Department of Animal Biosciences, University of Guelph, Guelph, ON, Canada N1G 2W1; Trouw Nutrition R&D, Boxmeer, the Netherlands; Department of Animal Biosciences, University of Guelph, Guelph, ON, Canada N1G 2W1

**Keywords:** α-linolenic acid, delayed-type hypersensitivity, keyhole limpet hemocyanin, n-6:n-3 ratio

## Abstract

*Camelina sativa* is an oilseed crop rich in α-linolenic acid (**ALA**), an n-3 fatty acid (**FA**), and is resistant to harsh climates and pests. Previously, supplementation with camelina oil (**CAM**) in horses had no adverse effects on basic health parameters and had comparable skin and coat parameters as both flaxseed oil (**FLX**) and canola oil (**OLA**). Further, the plasma FA profile of horses was reflective of their respective treatment oil. The objective of this study was to assess the effects of dietary CAM supplementation on skin FA profile, immune, and inflammatory responses as compared to 2 commonly used oils in the equine industry, OLA and FLX, in healthy adult horses. Twenty-four adult horses, from 2 separate herds, were enrolled in this experiment. The horses underwent a gradual 4-wk fat acclimation period to sunflower oil (approximately 0.28% ALA), then were supplemented with either CAM (approximately 34.9% ALA), OLA (approximately 12.0% ALA), or FLX (56.0% ALA) at an inclusion rate of 0.37 g/kg body weight (**BW**) per day for an additional 16 wk. Immune and inflammatory responses were assessed by measuring antibody concentrations across time after sensitization to keyhole limpet hemocyanin (**KLH**) at weeks 10 and 12, and a subsequent delayed-type hypersensitivity (**DTH**) challenge. Skin biopsy samples were collected at weeks 0, 8, and 16, and FA composition was determined using gas-chromatography. All data were analyzed as a repeated measures ANOVA using PROC GLIMMIX in SAS. Antibody and DTH responses to KLH did not differ among groups (*P* = 0.262 and 0.813, respectively), and no treatment by time effects were observed (*P* = 0.764 and *P* = 0.817, respectively). Most FA in the skin changed in composition across time, with the sum of n-3 FA increasing (*P* < 0.001) and the sum of n-6 FA and skin n-6:n-3 ratio decreasing over time (*P* < 0.001 and *P* < 0.001, respectively). Only dihomo-γ-linolenic acid (*P* = 0.025) and the sum of n-3 FA (*P* = 0.031) had treatment-by-week effects. At week 16, the composition of eicosapentaenoic acid in the skin was greater in FLX than OLA, but neither differed from CAM (*P* = 0.049). These results suggest that ALA supplementation may beneficially impact skin FA profile. However, due to the small differences in n-3 FA and n-6:n-3 ratio among CAM, FLX, and OLA, a comparable skin FA profile, immune, and inflammatory response was observed among treatments at a dose of 0.37 g oil/kg BW. Therefore, CAM may be a suitable alternative to FLX in equine diets for the delivery of ALA.

## Teaser

Camelina oil is an emerging alternative to other oils commonly supplemented with horses as a source of α-linolenic acid (**ALA**). The effects of camelina oil on skin fatty acid profile, immunity, and inflammation in horses were similar to those of flaxseed and canola oils, but the switch from an n-6 enriched oil to the ALA-rich treatment oils increased n-3 and decreased n-6 fatty acids and the n-6:n-3 ratio of the skin.

## Introduction

Supplementation with n-3 fatty acids (**FA**), including the plant-based α-linolenic acid (**ALA**) and marine-derived eicosapentaenoic acid (**EPA**) and docosahexaenoic acid (**DHA**), plays a role in supporting immunity and the balance between the need for inflammatory responses and resolution of that inflammation through anti-inflammatory responses. Many mechanisms have been investigated across species, including improving skin barrier function (De Spirt et al., 2008; Neukam et al., 2011; Barcelos et al., 2015), altering B cell populations and activation (Gurzell et al., 2013; Teague et al., 2013), increasing antibody production (Prickett et al., 1982; Kelley et al., 1988; Fritsche et al., 1991; Bazinet et al., 2004; Teague et al., 2013), and promoting macrophage polarization to the M2 tissue repair state (Titos et al., 2011; Chang et al., 2015; Cai et al., 2018). The n-6 FA linoleic acid (**LA**) and ALA compete for the same enzymes for conversion to precursors of inflammatory and anti-inflammatory eicosanoids, respectively (Brenner and Peluffo, 1966). In horses, the tissue and circulating FA profile is often a reflection of the FA profile of the diet consumed (O’Connor et al., 2007; Vineyard et al., 2010; Hess et al., 2012; Pearson et al., 2022). Therefore, altering the intake of ALA, LA, or their metabolites, has the potential to alter tissue FA profile and impact immunity and inflammatory responses as some of the FA are precursors of inflammatory mediators.

The FA profile of the skin directly impacts its physiological function of the skin (Kendall and Nicolaou, 2013). The beneficial impacts of n-3 FA supplementation on skin barrier function (De Spirt et al., 2008; Neukam et al., 2011; Barcelos et al., 2015) suggest a possible connection between skin FA profile and barrier function. Further, many types of the skin cells contain receptors involved in eicosanoid signaling (Hussain et al., 1994; Janssen-Timmen et al., 1995; Keeney et al., 1998; Abd‐El‐Aleem et al., 2001), indicating that FA present in the skin plays a role in immune and inflammatory responses. Further, the serum FA composition was observed to be different than the skin FA composition in horses, specifically, the skin had a greater composition than serum of n-3 and a lower composition of n-6 FA (Bergero et al., 2002). Skin also had a lower n-6:n-3 ratio than serum (Bergero et al., 2002). While studies in horses have investigated circulating FA profile, immunity, and inflammation in horses in response to ALA supplementation (Hall et al., 2004b; Vineyard et al., 2010), the alteration of the skin FA profile following ALA supplementation and associated changes in immune and inflammatory responses have not yet been investigated.

As most forages are abundant in ALA and have a low n-6:n-3 FA ratio, horses maintained on a forage-based diet typically consume a low n-6:n-3 ratio (Glasser et al., 2013). Conversely, most concentrates contain greater LA than ALA as reviewed by Burron et al. (2024). Thus, when access to good quality forages is limited, or horses are maintained on a concentrate-based diet, ALA intake may be reduced. In these cases, supplemental oils may be added to the diet to increase ALA intake and reduce the n-6:n-3 ratio. Canola oil (**OLA**) and flaxseed oil (**FLX**) are common supplemental oils used in the equine industry, with FLX being abundant in ALA and containing a low n-6:n-3 ratio (approximately 1:4; Olomu and Baracos, 1991; Eidhin et al., 2003; Teh and Birch, 2013; Mungure and Birch, 2014; Rueda et al., 2014). Camelina oil (**CAM**), produced from the oilseed crop *C. sativa*, has been investigated as a potential alternative for biofuels (Vollmann and Eynck, 2015) and for the delivery of ALA in horses (Burron et al., 2023; Richards et al., 2023). The crop itself is resistant to harsh climates, pests, and diseases that commonly affect other oilseed crops (Vollmann and Eynck, 2015). Further, the oil produced has a low n-6:n-3 ratio (approximately 1:2) and has a greater shelf life than FLX owing to its comparatively high tocopherol and polyphenol content, and lower n-3 content resulting in reduced susceptibility to rancidity (Eidhin et al., 2003; Ratusz et al., 2018). Additionally, when supplemented to healthy adult horses at a dose of 0.37 g oil/kg BW for 16 wk, no adverse effects were observed on basic health parameters including body weight (**BW**), body condition score (**BCS**), complete blood count and serum biochemistry of healthy adult horses (Burron et al., 2023).

The objective of the present study is to compare the effects of CAM (n-6:n-3 = approximately 1:2), FLX (n-6:n-3 = ~1:4), and OLA (n-6:n-3 = ~1:0.5) supplementation on the skin FA profile, and immune and inflammatory responses to the antigen keyhole limpet hemocyanin (**KLH**) in horses. We hypothesize that due to the increased ALA content in both FLX and CAM (55 and 35%, respectively; Table 1) compared to OLA (16%), horses supplemented with FLX or CAM will have an enhanced immune response and reduced inflammatory response during an immune challenge when compared to horses supplemented with OLA. Additionally, we hypothesize that the skin FA profile reflects the FA profile of the oil consumed but will contain greater n-3 and lower n-6 than the circulating FA profile previously reported by Burron et al. (2023).

**Table 1.** Predicted and analyzed fatty acid % composition of the sunflower, canola, flax, and camelina oil used in the equine feeding trial

Fatty acid, % composition	Sunflower oil^1^	Canola oil^2^	Flax oil^3^	Camelina oil^4^
16:0	5.00	4.39	5.91	5.50
16:1n-7	—	0.23	0.20	0.00
18:0	3.25	1.47	3.12	2.55
18:1 *cis*-9	53.3	57.7	17.2	14.0
18:1n7	—	—	0.89	1.02
18:2n-6	38.0	22.7	16.0	17.2
18:3n3	0.28	12.0	56.0	34.9
20:0	0.10	0.51	0.33	1.53
20:1n-9	0.04	1.01	0.33	15.8
20:1n-7	—	—	—	0.71
20:2n-6	—	—	—	2.14
20:3n-3	—	—	—	1.53
22:1n-9	—	0.10	—	3.16
Unsaturated fatty acids	91.6	93.6	90.6	90.4
SFA	8.38	6.37	9.36	9.57
MUFA	53.4	59.0	18.6	34.6
PUFA	38.2	34.6	72.0	55.8
n-6	38.0	22.7	16.0	19.3
n-3	0.28	12.0	56.0	36.5
n-9	53.4	58.8	17.5	32.9
n-7	0.00	0.23	1.10	1.73
n-6/n-3 ratio	135	1.89	0.29	0.53

Abbreviations: SFA = saturated fatty acids; MUFA = monounsaturated fatty acids; PUFA = polyunsaturated fatty acids.

^1^Numerical values represent average analytical values adapted from Rueda et al. (2014).

^2^Numerical values represent average analytical values adapted from Teh and Birch (2013), Ghazani et al. (2014), Mungure and Birch (2014).

^3^Numerical values represent average analytical values adapted from Olomu and Baracos (1991), Teh and Birch (2013), Mungure and Birch (2014), Rueda et al. (2014).

^4^Gas chromatography analysis by Smart Earth Camelina Corp. (Saskatoon, Saskatchewan, Canada). Table from Burron et al. (2023).

## Material and Methods

### Animals

All experimental procedures were approved by the University of Guelph’s Animal Care Committee (Animal Use Protocol #4481) and were carried out in accordance with national and institutional guidelines for the care and use of animals. Twenty-four adult horses (20 mares and 4 geldings) with an age of 14.2 ± 4.8 (mean ± SD) yr and a mean BW of 518 ± 63 kg (mean ± SD) were enrolled in this study. Horses had a median BCS of 6.5 (on a 9-point scale, range 5–8.5) and had no abnormalities on physical assessments, serum biochemistry profiles, or complete blood counts. Additionally, horses enrolled in this study were not receiving any anti-inflammatory medications. The study and sample collection were performed from June 2021 to January 2022, as previously described by Richards et al. (2023). Horses were housed outdoors in mixed herds in one of 2 locations: The University of Guelph Arkell Research Station (*n* = 21; Arkell, ON, Canada) and the University of Guelph Equine Sports Medicine and Reproduction Centre (**ESMRC**; Guelph, ON, Canada; *n* = 3). The study periods for the Arkell Research Station and ESMRC herds were June 2021 to December 2021 and August 2021 to January 2022, respectively. Locations differed slightly in management, where horses at the Arkell Research Station (*n* = 21) were on pasture (Tables S1 and S2) with no access to hay from June 2021 to the beginning of October 2021, then supplemented with ad libitum hay (Table S3) until mid-November 2021. These horses were re-located at this time to a winter barn on the same property where they were housed in outdoor paddocks with access to a large indoor space and ad libitum hay for the remainder of the study period. The hay provided at the winter barn was from the same source as the hay provided while on pasture. Horses at the EMSRC (*n* = 3) were provided ad libitum access to hay and intermittent access to pasture but were not re-located during the study period (August 2021—January 2022). Start dates for the feeding trial were staggered due to the availability of the horses, however, treatments were equally dispersed across locations and start dates.

### Study design and experimental treatments

In brief, horses underwent a 4-wk wash-in period where they were fed an increasing dose of sunflower oil until reaching a final inclusion level of 0.37 g oil/kg calculated BW. Sunflower oil was gradually increased at a rate of 0.05 g oil/kg BW The BW of horses was calculated by using the following formula (Carroll and Huntington, 1988):


$$\begin{array}{l} BW\;(kg)=\;\frac{Heart\;\;\;Girth\;\;\;(cm{)}^{2}\times Body\;\;\;Length\;\left(cm\right)\;}{11,877} \end{array}$$

Oil was mixed with timothy hay cubes (Premium Timothy Hay Cubes, Ontario Dehy, Goderich, ON, Canada) that were soaked to form a mash and fed either once or twice a day, depending on the location, start date, and existing management and feeding schedule of the facility. Horses were balanced by location, age, and BW, and then randomly assigned to one of 3 treatment oils: FLX (*n* = 8), CAM (*n* = 8), or OLA (*n* = 8). A negative control was not included as the main objective of the study was to compare CAM to the common supplemental oils FLX and OLA. After the 4-wk wash-in period, each horse’s assigned treatment oil replaced the sunflower oil and was incorporated into the diet in the same manner as the sunflower oil at an inclusion level of 0.37 g oil/kg calculated BW, in addition to free-choice forage for 16 wk.

### Sample collection and storage

#### Skin punch biopsies.

Skin punch biopsies were collected on weeks 0, 8, and 16. A small area of hair on the left side of the upper neck was clipped (Wahl Professional Pro Ion Clippers, #10 blade) and a 1 mL subcutaneous injection of 2% lidocaine (Teligent Inc., Mississauga, ON, Canada) was administered at the area of collection. The area was not sanitized or cleaned in order to not disrupt the skin sample. A 6 mm punch biopsy tool (Disposable Biopsy Punches, Integra Miltex #21909-144, Princeton, NJ, USA) was rotated clockwise with slight pressure until inserted into the subcutaneous tissue. Using tweezers and scissors, the skin sample was extracted, rinsed with saline, and blotted to dry using sterile gauze. The sample was placed into a microcentrifuge tube, snap-frozen in liquid nitrogen, and stored at −80 °C until analysis. The site of sample collection was cleaned with iodine, rinsed with saline, and closed using 2-3 Monocryl sutures (Ethicon 4-0 Monocryl P-3 Needle 18” Undyed Monofilament, Ethicon Inc., Raritan, NJ, USA). An antiseptic wound dressing (Dr. Naylor’s Red-Kote Aerosol Spray, Dr. Naylor, Morris, NY, USA) was applied topically to the area.

#### Keyhole limpet hemocyanin.

On weeks 10 and 12, injections of 500 μg KLH and 1 g of a saponin-based adjuvant (Quil-A, Croda International Plc., East Yorkshire, United Kingdom) were administered intramuscularly to each horse’s neck to stimulate a systemic immune response. Blood samples were collected prior to each injection (weeks 10 and 12) and on weeks 8, 14, and 16 via jugular venipuncture. Whole blood (5 mL) was collected into sodium heparin vacutainers (Becton, Dickinson and Company, Franklin Lakes, NJ, USA) using a 21-gauge multi-draw needle (Greiner Bio-One Multi-Drawing Blood Collection Needles, 21-gauge, 1.5 inch). Vacutainers were inverted 8 to 10 times immediately after collection and placed on ice until the end of blood collection. Samples were centrifuged at 3,500 × *g* for 15 min at room temperature (Fisherbrand accuSpin Micro 17 Microcentrifuge, Thermo Fischer Scientific, Waltham, MA, USA). Plasma was then separated, aliquoted, and frozen at −80 °C until KLH immunoassay analysis.

#### Delayed-type hypersensitivity test.

**Figure 1.**
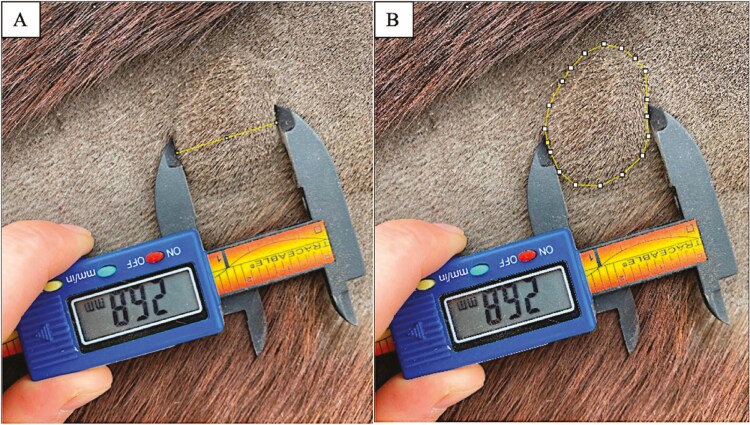
Example of how area of swelling after the DTH challenge performed after a series of 2 intramuscular KLH injections on weeks 10 and 12 in horses (*n* = 23) supplemented with either CAM, FLX, or OLA where (A) the scale was set by drawing a line from one end of the calipers to the other and inputting the displayed measurement, and (B) outlining the area of swelling using the polygon function in ImageJ (National Institutes of Health).

To evaluate cell-mediated immunity and localized inflammatory response, a delayed-type hypersensitivity (**DTH**) test was performed as previously validated by Engstrom et al. (2009). One day after the KLH injection was given on week 12, a large rectangular area of hair was clipped from the right side of each horse’s neck using a #10 blade. At equal spacing, an intradermal injection of 0.15 mL physiological saline as a negative control, 0.15 mL histamine (0.025 g/L; MilliporeSigma Histamine, Free Base, 97% Calbiochem #51-45-6) as a positive control, and 0.15 mL KLH (0.8 mg; heat aggravated in water bath at 70 °C for 30 min) was given within the clipped area. Induration reactions for all injection sites were observed and photos were taken using an iPhone XR (Apple Inc., Cupertino, CA, USA) at 0.5-, 1-, 8-, 24-, 48-, 72-, and 96-h postinjection for analysis. A digital caliper was included in the frame in each photo (Figure 1).

### Sample analysis

#### Fatty acid analysis.

Skin punch biopsy samples were thawed on ice and any subcutaneous fat (white tissue) was trimmed and discarded. To ensure consistency, only one person performed this step. The remaining skin was weighed and then homogenized in 0.1 M KCl (Sigma-Aldrich, Oakville, ON, Canada). The homogenate was transferred to a glass tube and a chloroform–methanol mixture (2:1; Thermo Fisher Scientific) was added to the tube. The homogenate mixture was rinsed with nitrogen, the glass tube sealed, and the mixture was left to incubate at 4 °C overnight. The next day, samples were centrifuged at 357 × *g* for 10 min at room temperature (21 °C; Sorvall Legned RT+, Thermo Fisher Scientific). The lower chloroform layer was extracted and dried down completely under nitrogen, after which they were reconstituted with 100  μ L chloroform (Thermo Fisher Scientific).

From the reconstituted sample, 15 µL was transferred to separate glass tubes to be used for total lipid analysis. To these samples, 10  μ L of C17:0 and 2 mL 0.5 M KOH in methanol (Thermo Fisher Scientific) were added to each sample. Samples were capped to prevent evaporation, vortexed for 5-10 s, and incubated at 100 °C for 1 h to saponify. Samples were then cooled at room temperature for 10 min. Once cooled, 2 mL hexane and 2 mL of 14% BF_3_-MeOH solution (Sigma-Aldrich) were added to each sample. Samples were capped to prevent evaporation, vortexed, and incubated at 100 °C for 1 h to methylate. Samples were cooled at room temperature for 10 min, after which 2 mL of double distilled water was added to each sample to stop methylation and samples were vortexed. Samples were centrifuged at 357 × *g* for 10 min at 21 °C. The top hexane layer of each sample was extracted and transferred into a clean gas-chromatography vial. Samples were dried down completely under nitrogen gas and then reconstituted with 100 µL of hexane (Sigma-Aldrich) for gas-chromatography analysis. Fatty acid methyl esters (**FAME**) were separated using the Agilent 7890-A gas chromatograph containing an SP-2560 fused silica capillary column (Sigma-Aldrich). The FA peaks were identified using OpenLab CDS EZChrome (Agilent, Edition A.04.06 version 1.255.227, 2014) by comparing peaks to FAME standards (NuCheck, Elysian, MN, USA). The percentage of FA in the skin was determined by using the following equation:


$$\begin{array}{l} \%\;FA=\frac{FA\;area}{Total\;area}\times 100 \end{array}$$

#### KLH immunoglobulin G immunoassay.

To evaluate humoral immunity, the KLH antibody response was evaluated based on an indirect enzyme-linked immunosorbent assay (**ELISA**) protocol outlined by Sturgill and Horohov (2010). Briefly, KLH protein (Sigma-Aldrich) was diluted in carbonate-bicarbonate buffer (Sigma-Aldrich) to create a 10 μg/mL coating solution and 96-well ELISA plates were coated by pipetting 100 µL of the solution to each well. The ELISA plates were sealed and incubated at 4 °C overnight. The plates were washed twice with a solution of 0.05% Tween-20 (Sigma-Aldrich) in phosphate-buffered saline (**PBST**; Sigma-Aldrich). The plates were then blocked by pipetting 100 µL of a solution of 1% fish gelatin (Sigma-Aldrich) in phosphate-buffered saline (**PBS**) into each well, sealed and incubated at room temperature for 1 h. Following incubation, the plates were washed twice with PBST. Plasma samples were diluted 1:8,000 in PBS. A negative control was created by pooling all samples from weeks 8 and 10 (prior to KLH vaccination) and diluted 1:8,000 in PBS. A reference sample was created by pooling all samples from weeks 12, 14, and 16 (after the KLH injections) and diluted 1:8,000 in PBS. Samples and controls were assayed in duplicate, with 100 μL of diluted sample or control added to the appropriate well. Samples were not added to blank wells. The plates were sealed and incubated for 1 h at 37 °C, then the plates were washed twice with PBST. Goat anti-horse immunoglobulin G (**IgG**) peroxidase secondary antibody solution (Jackson ImmunoResearch Laboratories Inc, West Grove, PA, USA) was diluted 1:10,000 in PBST and pipetted (100 µL) into each well, excluding blank wells. The plates were sealed and left to incubate for 1 h at 37 °C. The plates were washed twice with PBST and 100 µL of 3,3,5,5-tetramethylbenzidine substrate (Sigma-Aldrich) was added to each well. The plates were sealed and incubated at room temperature for 5 min. Then, 100 µL of tetramethylbenzidine stop reagent (Sigma-Aldrich) was added to each well and the plates were immediately read at 450 nm.

Intraplate CV was calculated shortly after results were obtained, and any samples with an intraplate CV of greater than 10% were run again on another plate on the same day. Since no standard existed for KLH antibody concentration in horses, results were left as optical density (**OD**) values. An increase in OD value represents an increase in KLH antibody. The OD value of the blank well was subtracted from the OD value of all other wells. To standardize OD values of samples across plates, the mean of the reference sample was corrected to an OD value of 1 and each sample OD value was then multiplied by the inverse mean of the reference sample to create corrected OD values for analysis.

One data point from week 16 was removed from the analysis as the OD result was lower than the blank well OD. One horse’s data from the CAM group was removed from both immune and inflammatory analysis due to an abnormal antibody response to KLH. At weeks 8 and 10 (prior to the first KLH injection), the horse had unusually high antibodies to KLH and a muted response to both the first and second KLH injection compared to the other horses. Therefore, data analysis for the KLH antibody and DTH response were performed with *n* = 23 horses.

#### DTH reaction measurements.

Pictures of injection sites were analyzed by the computer software ImageJ (National Institutes of Health, Bethesda, MD, USA) to determine the area of the reaction. Digital calipers (Fisherbrand Traceable Digital Carbon Fiber Calipers Catalogue #S90187A) were included in the frame of each photo, and using ImageJ, a scale (mm) was manually determined using the calipers as a reference. This was done by drawing a line from one end of the measuring device to the other and entering the distance (mm) displayed on the screen of the measuring device (Figure 1A). Then, using the polygon sections function in ImageJ, a line was drawn around the area of swelling surrounding the injection site (Figure 1B). Once the entire area of the reaction was outlined, the area (mm^2^) was automatically calculated by ImageJ using the pixels in the photo and the scale set previously. All injection site pictures were analyzed by a single operator. Any reaction larger than the negative control (saline) was considered to be a positive reaction. If a reaction to saline occurred, the area of induration from the saline injection was subtracted from the area of induration from the KLH injection. The negative control helped to rule out other causes for swelling around the injection site including trauma from the injection itself as well as the volume of the injected substance.

### Statistical analysis

Statistical analyses of the data were performed using PROC GLIMMIX in SAS Studio software (v.9.4, SAS Institute Inc., Cary, NC, USA). The horse was the experimental unit and the treatment oil type (CAM, OLA, and FLX) was treated as a fixed effect. Week (0, 8, 10, 12, 14, 16) was treated as a repeated measure, and location was treated as a random effect and retained when significant. An ANOVA was performed to assess the effects of treatment (**TRMT**), time (**WEEK** or **TIME**), and their interaction on FA % composition, area of swelling around injection site, and relative KLH antibody concentration. Assumptions of normality were evaluated using the Shapiro–Wilk normality test and by visual assessment of the residuals. For both the KLH antibody response and DTH area of swelling data, as well as some of the FA data, the residuals were not normally distributed. As such, data were log-transformed. Back-transformed least-square means were used to assess differences in means of treatment, week, time, treatment-by-week, and treatment by time interactions. Significance was declared at a *P < *0.05 and trends were declared at *P* ≤ 0.10. When fixed effects were significant, means were separated using a Tukey–Kramer Adjustment. Results are presented as least-square means ± 95% confidence intervals.

## Results

### Skin FA

**Figure 2.**
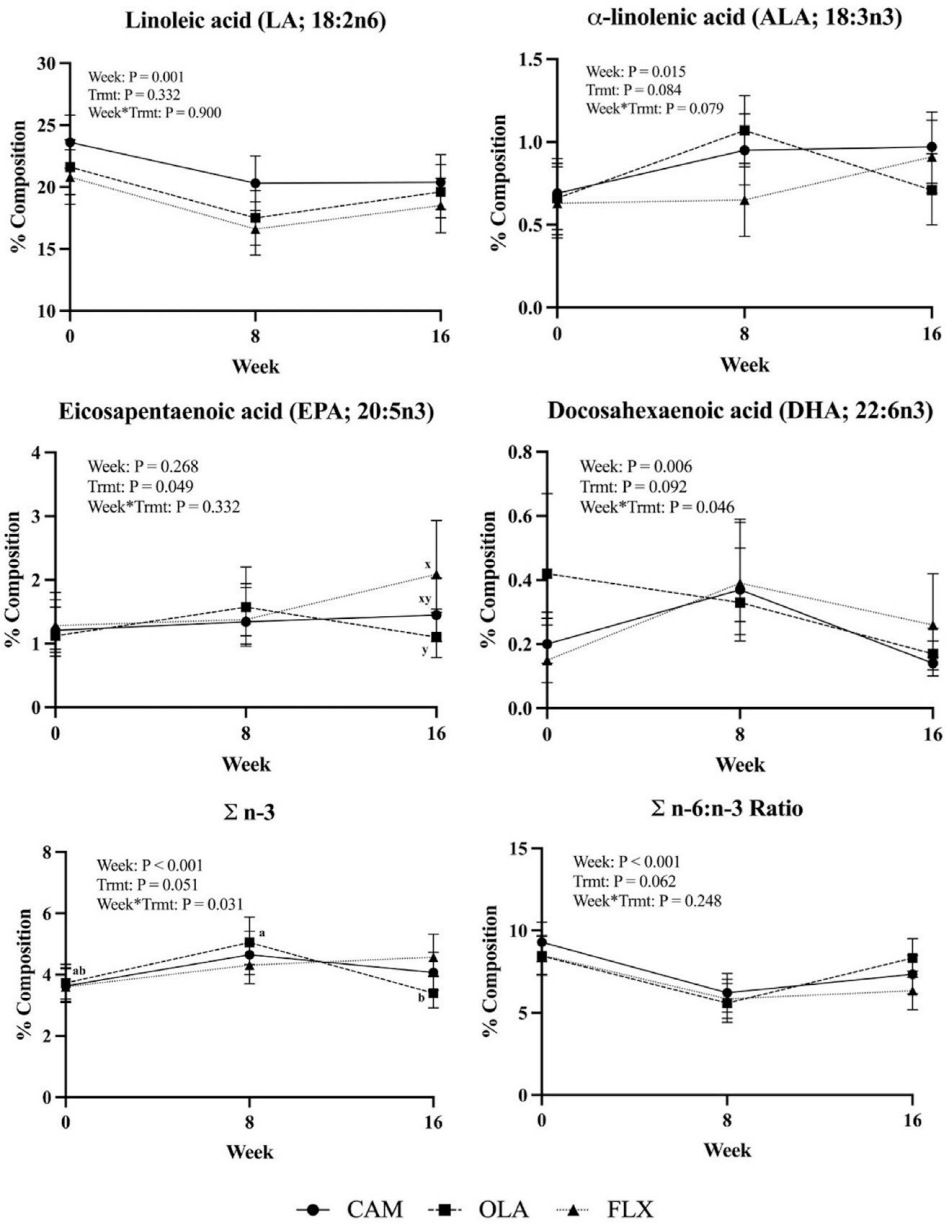
Select skin fatty acid % composition (mean ± 95% confidence intervals) in horses (*n* = 24) supplemented with either CAM, OLA, or FLX across 16 wk. Letters a,b indicate differences across time for a particular treatment (Trmt) group (*P* < 0.05). Letters x,y indicate differences among treatments at week 16 (*P* < 0.05).

All FA identified in the skin are reported in Table S4 and select FA are illustrated in Figure 2, however, only specific FA relevant to the scope of this study will be discussed in the results and discussion. Across all treatments and time points, the predominant FA (>10% composition) in the skin were LA (18:2n6), stearic (18:0), palmitic (16:0), and oleic acid (18:1c9). Further, saturated FA (**SFA**) were the most abundant FA group (approximately 45%), followed by polyunsaturated FA (**PUFA**; approximately 34%), then monounsaturated FA (**MUFA**; approximately 20%) and n-6 FA (approximately 28 %) composition was greater than n-3 FA (approximately 4%).

There were no TRMT or WEEK by TRMT interactions for SFA content of the skin (*P* = 0.954 and *P* = 0.842, respectively). There were several WEEK effects on SFA content of the skin when treatments were pooled, where for example stearic acid (18:0) was greater in week 0 than week 8, but neither differed from week 16 (*P* = 0.003). However, the sum of SFA did not differ across weeks (*P* = 0.366). There were no TRMT or WEEK by TRMT interactions for MUFA (*P* = 0.285 and *P* = 0.445, respectively). The sum of all MUFA did not differ across weeks (*P* = 0.282).

There was a WEEK by TRMT interaction for dihomo-γ-linolenic acid (**DGLA**; 20:3n6; *P* = 0.025) whereby the percent DGLA in the FLX treatment was greater at weeks 0 and 16 than week 8. The sum of n-3 FA had a WEEK by TRMT effect (*P* = 0.031), where for the OLA treatment the sum of n-3 was greater in week 8 than in week 16, but neither week 8 nor week 16 differed from week 0. While DHA (22:6n3) showed a WEEK by TRMT effect (*P* = 0.046), no differences were observed after means were separated. No other PUFA showed WEEK by TRMT effects (*P* > 0.050). Removing the WEEK < 16 data from the model and analyzing only week 16, only EPA had a TRMT effect (*P* = 0.049). The % composition of EPA (*P* = 0.049) was greater in FLX than OLA, but neither FLX nor OLA differed from CAM. There was a trend for ALA (*P* = 0.084) to be greater in CAM than OLA and for DHA (*P* = 0.092) to be greater in FLX than in CAM. Additionally, there was a trend for the sum of n-3 FA (*P* = 0.051) to be greater and the n-6:n-3 ratio (*P* = 0.062) to be lower in FLX than OLA. All other FA were similar among treatments at week 16 (*P* > 0.100).

Several PUFA showed a WEEK effect when data was pooled across treatments. The % composition of LA (18:2n6; *P* = 0.001) was greater in week 0 than in weeks 8 and 16, but LA did not differ between weeks 8 and 16. A similar pattern was observed for arachidonic acid (20:4n6; *P* = 0.007), where % composition was greater in week 0 than weeks 8 and 16, but weeks 8 and 16 did not differ. The % composition of DGLA (*P* = 0.002) was also lower in week 0 as compared to week 8 but week 0 did not differ from week 16. There was a trend for the % composition of DGLA to be greater in week 16 than in week 8. The opposite was seen in ALA (18:3n3; *P* = 0.015), whereby % composition of ALA was greater in weeks 8 and 16 than in week 0, but ALA did not differ between weeks 8 and 16. The % composition for docosapentaenoic acid (DPA; 22:5n3; *P* = 0.001) was lower in week 8 compared to weeks 0 and 16 but did not differ between weeks 0 and 16. Docosahexaenoic acid (DHA; 22:6n3; *P* = 0.006) was greater in week 8 than in week 16, but neither differed from week 0. The % composition of the sum of PUFA (*P* = 0.010) was greater in week 0 than weeks 8 and 16 but did not differ between weeks 8 and 16. Similarly, the sum of n-6 FA (*P* < 0.001) were greater in week 0 than in weeks 8 and 16 but did not differ between weeks 8 and 16. The sum of n-3 FA (*P* < 0.001) were greatest in week 8 compared to weeks 0 and 16 but did not differ between weeks 0 and 16. The ratio of n-6 to n-3 FA (*P* < 0.001) was greater in week 0 than in weeks 8 and 16 and greater in week 16 than in week 8. It should be noted that the WEEK effects for DGLA and n-3 are likely influenced by FLX and OLA, respectively, as seen in the WEEK by TRMT effects. The % composition of all other PUFA were similar among weeks (*P* > 0.050).

### KLH antibody response

The inter-plate CV for all ELISA plates, based on the positive and negative controls, and intraplate CV for all samples, were <10% with the exception of 2 data points that were removed from the data set.

**Figure 3.**
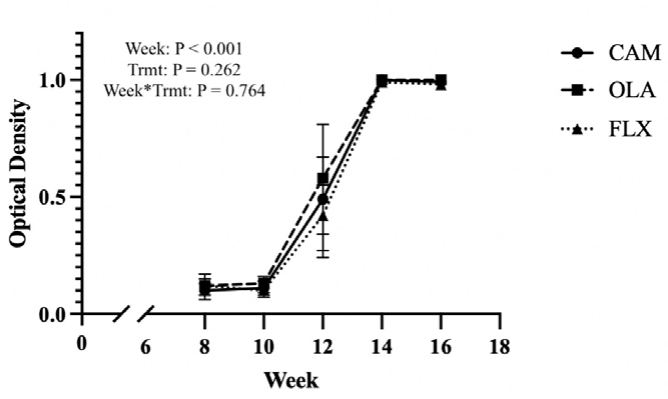
Mean corrected OD values (± 95% confidence intervals) representing relative antibody concentration in horses (*n* = 23) supplemented with CAM, FLX, or OLA across 16 wk during an immunization challenge whereby horses received an intramuscular injection of KLH on weeks 10 and 12.

Corrected OD values for treatment groups across WEEK can be seen in Figure 3. Relative antibody concentrations did not differ among treatments (*P *= 0.262) or have a WEEK by TRMT effect (*P =* 0.764) but did differ across weeks when samples were pooled among treatments (*P < *0.001). Relative antibody concentration was not different between weeks 8 and 10 but increased at week 12. A further increase was observed at week 14. The KLH immune challenge was successful in eliciting a typical primary and secondary antibody response in all but one horse, whereby antibody production increases after the primary vaccine and further increases after the booster vaccine (Janeway et al., 2001). The full medical history of the horses enrolled in this trial was not available, thus it is possible that the horse that did not have a typical antibody response had been previously exposed to KLH causing the high concentration of KLH antibodies at baseline. Additionally, while all horses were deemed healthy upon enrollment based on basic hematological and blood chemistry analysis, we cannot exclude the possibility of an underlying health condition resulting in the muted response to KLH.

### DTH skin response

**Figure 4.**
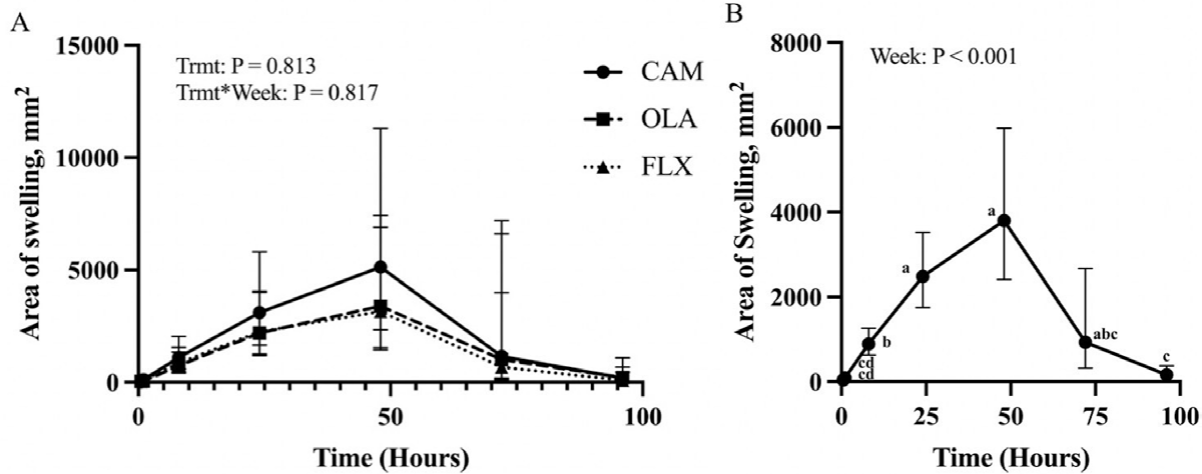
Mean area of swelling (mm^2^± 95% confidence intervals) around the KLH injection site during the DTH challenge performed after a series of 2 intramuscular KLH injections on weeks 10 and 12 in horses (*n* = 23) (A) supplemented with CAM, FLX, or OLA, and (B) pooled across treatments. Letters a,b,c,d indicate differences across time for a particular treatment group (*P* < 0.05).

The KLH and histamine injections both produced greater induration reactions than saline (*P* < 0.001 and *P* = 0.003, respectively), and the KLH injection produced a greater induration reaction than the histamine injection (*P* < 0.001). The area of swelling around the KLH injection site across time for each treatment group is illustrated in Figure 4A. The mean area of swelling (mm^2^) did not differ among treatments (*P* = 0.813), and there were no TIME-by-TRMT interactions (*P* = 0.817). The area of swelling around the KLH injection site differed across time when pooled among treatments (*P* < 0.001; Figure 4B). The area of swelling was not different between 30- and 60-min postinjection but increased at 8 h postinjection. A further increase was observed at 24 h, and continued to increase numerically at 48 h, but the area of swelling was not different between 24- and 48-h postinjection. At 72 h, the induration reaction decreased numerically from 48 h and was significantly reduced at 96-h postinjection as compared to 48-h postinjection. At 96-h postinjection, the area of induration was not different than 30- and 60-min postinjection.

## Discussion

To the authors’ knowledge, this is the first study to examine the response of feeding different oils on the skin FA, immune, and inflammatory response in healthy adult horses. Switching horses from an n-6-enriched oil (sunflower) to ALA-enriched oils (CAM, FLX, and OLA) resulted in a decrease in LA content (%) and a decrease in the n-6:n-3 ratio of the skin. These effects were similar after 8 and 16 wk of feeding the treatment oils, suggesting that changes in the skin FA profile are achieved in under 8 wk of a change in the intake of FA. However, skin FA did not respond to the small differences in FA profile present among FLX, CAM, and OLA, although a greater number of horses may be required to detect these small differences in fatty acid concentrations.

In the present study, the increased content (%) of EPA in the FLX compared to OLA group suggests that some of the ALA from FLX was being converted to EPA and incorporated into the skin. The conversion of EPA from ALA is generally considered a slow and inefficient process in humans (Emken et al., 1990), but the efficacy of conversion is species-specific and has not been explored in horses. However, the incorporation of ALA metabolites, including EPA, into tissues following ALA supplementation may be suggestive of some conversion. This has been demonstrated in growing rabbits, where dietary supplementation with camelina cake (ALA-rich) resulted in increased EPA composition of the meat (Kokoskova et al., 2025). In horses fed milled flaxseed, skeletal muscle had increased DPA than non-supplemented horses (Hess et al., 2012), aligning with the increased EPA % composition of the skin following ALA supplementation observed in the current study, indicating that horses may have the ability to convert ALA to its longer chain metabolites.

The present study observed an increase in % composition of EPA in the skin of the FLX group compared to the OLA group, but no difference in ALA % composition. In the plasma of horses in this study, reported by Burron et al. (2023), ALA was greater in FLX than OLA, but EPA was not detected. Bergero et al. (2002) reported several differences in FA profile, including greater n-3 and reduced n-6 % composition of horse skin when compared to serum, indicating selective inclusion of FA in the skin and potentially differing FA responses to ALA supplementation. As plasma and serum FA profiles are comparable (Buchanan et al., 2021), the different response of the skin FA observed in the current study as compared to the plasma FA reported by Burron et al. (2023) could be at least partially due to underlying differences in plasma and skin compositions, though this was not statistically compared.

The literature surrounding skin FA profile in response to ALA supplementation is scarce, with only one study, to the author’s knowledge, in horses (Bergero et al., 2002). Unlike the current study, Bergero et al. (2002) observed no differences in the skin of horses supplemented with 0.047 g oil/kg BW of the ALA-rich Viper’s Burgloss oil (28.7% ALA) as compared to non-supplemented horses, though this was suspected to be due to the low dose of oil used compared to the current study. Further, dose-dependent increases in the incorporation of dietary FA into circulation have also been demonstrated in humans (Chan et al., 1993; Arterburn et al., 2006) and horses (King et al., 2008; Pearson et al., 2022). Thus, higher doses of ALA may elicit a greater magnitude of changes in the FA profile, both in circulation and in tissues. Aside from dose, the lack of treatment differences for other skin FA could also be due to the possible preferential incorporation of certain FA, such as ALA, LA, and ARA, into the skin (Schürer et al., 1994, 1999; Bowen and Clandinin, 2000). Interestingly, the present skin FA results, particularly EPA and ALA, appear to be more consistent with the results observed by Bazinet et al. (2004) and Hess et al. (2012) in the muscles of pigs and horses, respectively. This may suggest that the FA profile of the skin is similar to that of muscle, though this has not been investigated to the author’s knowledge.

The differences in dietary FA composition of FLX, CAM, and OLA did not result in the same skin FA response as the plasma FA response reported by Burron et al. (2023) despite being the same study. Particularly, some of the other skin FA increase or decrease in week 8, then return to baseline values. Since FA are presented here as % composition, changes to larger FA may have altered the % composition of smaller FA. Factors aside from diet may have also contributed to the skin FA profile changes over time. Human studies have observed alterations in the skin surface FA profile in response to season (Conti et al., 1996; Rogers et al., 1996), with some changes being positively correlated to skin temperature (Abe et al., 1980), suggesting n-3 FA may decrease in colder seasons. Horses in the present study were housed mainly outdoors and experienced a change in season part way through the trial (Fall to Winter), however the only change in FA over time that agrees with the human studies is the decrease in LA in the winter season as compared to summer (Conti et al., 1996; Rogers et al., 1996). It is possible that decreasing temperatures could have impacted the metabolic demands of the horses, thus changing the fate of some FA towards β-oxidation (Houten et al., 2016) rather than incorporation into the skin. Additionally, the majority of horses experienced a basal diet shift from pasture and hay to solely hay after relocation to a winter barn. As pasture typically contains a greater ALA content than hay (Glasser et al., 2013), the dietary shift mid-trial may have reduced overall n-3 intake, thus resulting in lower skin n-3 in week 16 than in week 8. Additionally, the KLH immune challenge may have also influenced the n-3 FA in the skin as during an immune response, FA are utilized to provide energy to drive immune cell function and act as precursors for immune molecules (Zivkovic et al., 2011; Zhou et al., 2023). Given the nature of horses, parsing these environmental effects on skin FA concentrations will be difficult and require iterative studies to tease these variables apart.

While FA play a role in numerous biological functions, their inflammatory and immune-modulating effects have been a main focus in literature. In the present study, the main n-3 FA provided in all 3 supplemented oils was ALA, and while the % composition of EPA in the skin differed, the immune response to KLH did not differ among treatments. Conversely, diets supplemented with a source of ALA altered the antibody production after stimulation with an antigen in rabbits, chickens, and pigs (Kelley et al., 1988; Sijben et al., 2001; Bazinet et al., 2004, respectively). Although, this is likely due to the lack of an n-6 negative, or low-fat/high starch control group in the present study. Indeed, the dietary differences in ALA content among treatments in the present study are relatively small compared to the differences in the studies by Kelley et al. (1988), Sijben et al. (2001), and Bazinet et al. (2004). Further, Sijben et al. (2001) investigated 16 diets varying in LA:ALA ratio ranging from approximately 29:1 to 2:1 and observed both diets with high LA:low ALA or high ALA:low LA, but not high ALA:high LA caused increased production of anti-KLH IgG antibodies in layer hens (Sijben et al., 2001). This is an interesting result as n-3 and n-6 FA are thought to have opposing effects on immune function, but could indicate that n-6:n-3 ratio may be a more important factor than the individual FA in altering antibody response. While the n-6:n-3 ratio of the skin was numerically different, the ratio was not statistically different among treatments likely owing to the study being underpowered, a common problem when conducting research in horses. It is possible that the difference in n-6:n-3 ratio of the oils was not different enough to elicit detectable differences in either the n-6:n-3 ratio of the skin and the immune response, especially when consuming a basal diet (i.e., forages) with an already low n-6:n-3 ratio (Glasser et al., 2013). Therefore, the similarities of treatment oils and the exclusion of an n-6 negative control group likely underpin the lack of differences in antibody response among treatments.

The immune-inflammatory response was also assessed by inducing a DTH reaction, which is a cell-mediated immune reaction characterized by a localized inflammatory response after exposure to an antigen (Vohr, 2005). This reaction is typically assessed by measuring the area of swelling or skin thickness around the injection site (Korver and Klasing, 1997; Bazinet et al., 2004; Vineyard et al., 2010). All horses on all dietary treatments exhibited a typical DTH response to the KLH antigen whereby the peak reaction occurred 24 to 72 h post-exposure to the antigen (Vohr, 2005). Similar to the immune response, the immune-inflammatory response was not different among treatment groups. There is a dearth of literature on the effects of dietary ALA supplementation on DTH response in horses. To the author’s knowledge, only one previous study exists investigating the DTH response in horses supplemented with ALA (milled flaxseed) compared to a non-supplemented group (Vineyard et al., 2010). The area of swelling was similar between the milled flaxseed and non-supplemented group at all time points, but the peak reaction occurred much more quickly than in the current study (Vineyard et al., 2010). Conversely, in pigs fed a high ALA diet, Bazinet et al. (2004) reported a greater % increase in DTH reaction to *Mycobacterium* 24-h postinjection as compared to pigs fed a low ALA diet. Regarding both immune and inflammatory responses, it is possible that different antigens elicit differing magnitudes of reaction, thus contributing to the variability in antibody and DTH response in literature and the current results. Species differences may also play a role, as some animals have been observed to have different immune cell genetics and antigen-specific sensitivities (Burrows, 1981; Tateda et al., 1996 cited in Karagianni et al., 2021). Overall, there appears to be variability in immune and inflammatory response following ALA supplementation in literature, though it is likely that these differences are due to a combination of factors including species differences, dose, and the magnitude of differences in n-6:n-3 ratio among treatments.

While EPA and DHA were detected in the skin of horses in the present study, the treatment oils supplied ALA, rather than EPA and DHA which are typically supplied through marine sources. The literature surrounding direct supplementation with EPA and DHA on antibody response in various species is mixed, with some reporting increased antibody production and activation with supplementation (Prickett et al., 1982; Fritsche et al., 1991; Weise et al., 2011; Ramon et al., 2012; Gurzell et al., 2013), and other reporting no impact on antibody response (Korver and Klasing, 1997; Wander et al., 1997; Hall et al., 2003, 2004a). Similarly, the DTH reaction has been observed to be both enhanced (Korver and Klasing, 1997; Hall et al., 2004a; Vineyard et al., 2010) or suppressed (Wander et al., 1997; Hall et al., 2003, 2004a) following fish oil supplementation, with some evidence of a dose–response (Korver and Klasing, 1997; Wander et al., 1997). As skin swelling is a sign of inflammation, the inconsistencies in DTH response to fish oil reported in the literature make it unclear whether fish oil enhances or suppresses the inflammatory response. While we did observe a greater composition of EPA in the skin of horses fed FLX as compared to OLA, similar to the small differences in n-6:n-3 ratio among treatment oils, there may not have been a large enough difference in EPA composition of the skin between the 2 groups to detect differences in immune and inflammatory responses. However, it is possible that with higher doses of ALA resulting in greater incorporation of EPA, different responses may be detected.

There were several limitations to the current study that may have impacted the results. The first limitation was the lack of a high n-6 negative control group, however, the objective of the present study was to compare CAM to similar oils rather than to a high n-6 oil. Additionally, due to the availability of horses, it was not possible to narrow the age range, breed, BW, or location of horses enrolled. With increasing age, changes in the immune system and function are observed in horses, often termed “inflamm-aging” (McFarlane et al., 2001, 2015; Horohov et al., 2002; Adams et al., 2008; Schnabel et al., 2015; Miller et al., 2021). Additionally, BW and BCS have also been related to immune changes, especially in aged horses (Vick et al., 2007; Adams et al., 2009). While treatments were balanced using these variables, they did increase the variability of the outcomes as horses were likely to experience different levels of inflammation. Moreover, horses were likely receiving sufficient ALA quantitates from the basal diet alone, were not enrolled in any training programs, and were healthy upon enrollment. Reduced ALA content in the basal diet, enrollment in an exercise program, and/or health concerns related to inflammation all could potentially increase ALA demands, thus influencing the effect of supplemental ALA and increasing differences among dietary treatments. Lastly, the variability in basal diet was also an uncontrollable limitation. Changes in FA profile of pasture over season and differences in pasture and hay intake among horses result in differences in FA consumption. Additionally, nutrient and FA composition of pasture and hay fed to horses at the ESMRC (*n* = 3) and FA composition of hay fed to horses at the Arkell Research Station were not evaluated.

## Conclusion

In summary, a switch from an n-6 enriched oil to an n-3 enriched oil (CAM, OLA, or FLX) resulted in increases in n-3 composition and decreases in n-6 composition and n-6:n-3 ratio of the skin within 8 wk of supplementation at a dose of 0.37 g oil/kg BW. Horses also showed the capacity to accumulate long-chain polyunsaturated FA in the skin, being sensitive to the dose of ALA to improve EPA and DHA concentrations. Further, horses supplemented with CAM, OLA, or FLX for 16 wk had similar skin FA profiles and immune and immune-inflammatory responses. Future studies should focus on comparing immune, inflammatory, and skin FA responses in a more focused population of horses, such as those experiencing inflammation. Further, they should explore supplementation with these 3 oils in horses receiving a basal diet with low ALA, or stressed models, such as exercise challenges, to further investigate differences in immunity and inflammation in response to n-3 FA supplementation. Nevertheless, supplementation with an ALA-enriched oil may be recommended to beneficially impact the skin FA profile by favoring incorporation of n-3 FA. Additionally, in healthy adult horses, CAM appears to be a suitable alternative to FLX or OLA in supporting the skin FA, immune and inflammatory response.

## Supplementary Material

skaf025_suppl_Supplementary_Materials

## Abbreviations

AA: arachidonic acid
ALA: alpha-linolenic acid
BW: body weight
CAM: camelina oil
DHA: docosahexaenoic acid
DTH: delayed-type hypersensitivity
EFA: essential fatty acid
ELISA: enzyme-linked immunosorbent assay
EPA: eicosapentaenoic acid
ESMRC: Equine Sports Medicine and Reproduction Centre
FA: fatty acid
FLX: flaxseed oil
IgG: immunoglobulin G
KLH: keyhole limpet hemocyanin
LA: linoleic acid
MUFA: monounsaturated fatty acid
OD: optical density
OLA: canola oil
PBS: phosphate buffer solution
PBST: Tween-20 in phosphate buffer solution
PUFA: polyunsaturated fatty acid
SFA: saturated fatty acid
