# Supplementary material for: The effect of dietary camelina, flaxseed, and canola oil supplementation on skin fatty acid profile and immune and inflammatory responses in healthy adult horses
Source: J Anim Sci. 2025 Feb 4;103:skaf025. doi: 10.1093/jas/skaf025 (PMC11897893; doi:10.1093/jas/skaf025)
Supplement: skaf025_suppl_Supplementary_Materials [file skaf025_suppl_supplementary_materials.docx]

**The effect of dietary camelina, flaxseed, and canola oil supplementation on skin fatty acid profile and immune and inflammatory responses in healthy adult horses**

Samantha Hartwig^†^, Scarlett Burron^†^, Taylor Richards^†^, Alexandra Rankovic^†^, David W.L. Ma^‡^, Wendy Pearson^†^, Jennifer Ellis^†^, Luciano Trevizan^$^, Dave J. Seymour^†,§^, Anna K. Shoveller^†1^

*Journal of Animal Science*

(Supplementary Materials S1, S2, S3, S4).

**Supplementary Table S1.** Fatty acid profiles (as-fed) of pasture grass consumed as the basal diet during a 16-week feeding period for horses consuming camelina, flaxseed, or canola oil (370 mg/kg BW/day) at Arkell Research Station.

|  | Pasture^1^ | |
| --- | --- | --- |
|  | June 8^th^ | July 6^th^ |
| Saturated Fatty Acids (%) | 0.400 | 0.520 |
| *cis*-Monounsaturated Fatty Acids (%) | <0.10 | <0.10 |
| *cis*-Polyunsaturated Fatty Acids (%) | 0.250 | 0.320 |
| n-3 Polyunsaturated Fatty Acids (%) | 0.170 | 0.220 |
| n-6 Polyunsaturated Fatty Acids (%) | <0.10 | 0.100 |
| Trans-Fatty Acids (%) | <0.10 | <0.10 |

^1^Pasture samples were collected by mixing 40 small “grab” samples from random locations in the field on the specified date. Samples were submitted to SGS Canada Inc., Agriculture and Feed for analysis. Horses (n=21) at Arkell Research Station, Ontario, Canada were consuming *ad libitum* pasture as the basal diet from June until October, when pasture was additionally supplemented with hay.

Table from Burron et al. (2023)

**Supplementary Table S2.** Nutrient analysis (dry matter) of pasture grass consumed as basal diet during a 16-week feeding period for horses consuming camelina, flaxseed, or canola oil (370 mg/kg BW/day) at Arkell Research Station.

|  | Pasture^1^ | |
| --- | --- | --- |
|  | June 8^th^ | July 6^th^ |
| Moisture (%) | 69.83 | 59.62 |
| Protein |  |  |
| Protein % (N x 6.25) | 10.84 | 8.84 |
| SP (%) | 3.12 | 1.97 |
| SP % of CP | 28.78 | 22.29 |
| ADF-CP % | 1.48 | 0.84 |
| ADF-CP as % of CP | 13.65 | 9.50 |
| NDF-CP% | 6.42 | 6.41 |
| NDF-CP as % of CP | 59.23 | 72.51 |
| Fibres |  |  |
| Acid Detergent Fibre (%) | 31.62 | 32.08 |
| aNeutral Detergent Fibre (%) | 53.17 | 56.47 |
| Lignin % | 4.80 | 5.36 |
| Non-Fibres |  |  |
| Ethanol Soluble CHO (%) | 5.94 | 6.87 |
| Water Soluble CHO (%) | 9.89 | 12.72 |
| Non-Structural Carbohydrates | 12.83 | 15.90 |
| Starch (%) | 2.94 | 3.18 |
| Fat (%) | 2.12 | 2.10 |
| Minerals |  |  |
| Ash % | 5.74 | 5.07 |
| Calcium (%) | 0.42 | 0.46 |
| Phosphorus (%) | 0.22 | 0.21 |
| Potassium (%) | 2.18 | 1.53 |
| Magnesium (%) | 0.21 | 0.23 |
| Sodium % | 0.04 | 0.01 |
| Copper (ppm) | 8.9 | 7.59 |
| Iron (ppm) | 117.29 | 108.83 |
| Manganese (ppm) | 50.62 | 49.06 |
| Zinc(ppm) | 27.44 | 23.36 |
| Ca:P Ratio | 1.91 | 2.19 |
| Zn:Cu Ratio | 3.08 | 3.08 |
| Energy (ADF Based) |  |  |
| TDN (%) | 50.39 | 48.48 |
| Digestible Energy (MCal/Kg) | 2.22 | 2.14 |
| Other |  |  |
| Relative Feed Value | 112.44 | 105.28 |

Abbreviations: N = nitrogen; SP = soluble protein; CHO = carbohydrate; TDN = total digestible nutrients.

^1^Pasture samples were collected by mixing 40 small “grab” samples from random locations in the field on the specified date. Samples were submitted to SGS Canada Inc., Agriculture and Feed for analysis. Horses (n=21) at Arkell Research Station, Ontario, Canada were consuming *ad libitum* pasture as the basal diet from June until October, when pasture was additionally supplemented with hay.

**Supplementary Table S3.** Nutrient analysis (dry matter) of hay consumed as basal diet during a 16-week feeding period for horses consuming camelina, flaxseed, or canola oil (370 mg/kg BW/day) at Arkell Research Station.

|  | Hay^1^ |
| --- | --- |
| Moisture (%) | 11.42 |
| Protein |  |
| Protein % (N x 6.25) | 12.39 |
| SP (%) | 3.61 |
| SP % of CP | 29.14 |
| ADF-CP % | 1.72 |
| ADF-CP as % of CP | 13.88 |
| NDF-CP% | 6.11 |
| NDF-CP as % of CP | 49.31 |
| Fibres |  |
| Acid Detergent Fibre (%) | 35.88 |
| aNeutral Detergent Fibre (%) | 58.10 |
| Lignin % | 5.74 |
| Non-Fibres |  |
| Ethanol Soluble CHO (%) | 5.56 |
| Water Soluble CHO (%) | 9.31 |
| Non-Structural Carbohydrates | 11.47 |
| Starch (%) | 2.16 |
| Fat (%) | 2.04 |
| Minerals |  |
| Ash % | 7.22 |
| Calcium (%) | 0.51 |
| Phosphorus (%) | 0.22 |
| Potassium (%) | 2.46 |
| Magnesium (%) | 0.19 |
| Sodium % | 0.01 |
| Copper (ppm) | 3.77 |
| Iron (ppm) | 69.92 |
| Manganese (ppm) | 39.12 |
| Zinc(ppm) | 23.34 |
| Ca:P Ratio | 2.27 |
| Zn:Cu Ratio | 6.19 |
| Energy (ADF Based) |  |
| TDN (%) | 48.23 |
| Digestible Energy (MCal/Kg) | 2.13 |
| Other |  |
| Relative Feed Value | 97.59 |

Abbreviations: N = nitrogen; SP = soluble protein; CHO = carbohydrate; TDN = total digestible nutrients.

^1^Large bale, 1^st^ cut (2020) hay from Arkell Research Station, Ontario, Canada. Average values of core samples from three bales in separate areas of storage. Samples were submitted to SGS Canada Inc., Agriculture and Feed for analysis. Horses (n=21) at Arkell Research Station, Ontario, Canada were provided hay *ad libitum* to supplement pasture consumption from October to November 2021, then consumed only hay *ad libitum* from November to December 2021 (end of study).

Table adapted from Burron et al. (2023)

**Supplementary Table S4.** The fatty acid % composition, presented as the least square mean ± 95% confidence interval, of the skin of healthy adult horses (n = 24) supplemented with either camelina (CAM), canola (OLA), or flaxseed oil (FLX) at a level of 0.37 g oil/kg BW per day for 16 weeks.

| **Fatty acid, %** | **Trmt^1^** |  | **Week** |  |  |  | **P-values** |  |
| --- | --- | --- | --- | --- | --- | --- | --- | --- |
|  |  | **0** | **8** | **16** |  | **Week** | **Trmt^2^** | **Week*Trmt** |
| 14:0 | CAM | 0.61 (0.53 – 0.72) | 0.70 (0.60 – 0.82) | 0.76 (0.65 – 0.89) |  | 0.038 | 0.578 | 0.976 |
|  | OLA | 0.64 (0.55 – 0.74) | 0.73 (0.62 – 0.87) | 0.73 (0.63 – 0.86) |  |  |  |  |
|  | FLX | 0.70 (0.60 – 0.82) | 0.76 (0.65 – 0.89) | 0.80 (0.68 – 0.93) |  |  |  |  |
|  | Mean | 0.65^b^ (0.59 – 0.71) | 0.73^ab^ (0.67 – 0.80) | 0.76^a^ (0.70 – 0.84) |  |  |  |  |
| 15:0 | CAM | 0.61 (0.51 – 0.73) | 0.71 (0.59 – 0.84) | 0.64 (0.53 – 0.76) |  | 0.324 | 0.248 | 0.774 |
|  | OLA | 0.68 (0.57 – 0.83) | 0.70 (0.58 – 0.85) | 0.60 (0.50 – 0.72) |  |  |  |  |
|  | FLX | 0.64 (0.54 – 0.76) | 0.76 (0.64 – 0.91) | 0.71 (0.60 – 0.85) |  |  |  |  |
|  | Mean | 0.65 (0.58 – 0.72) | 0.72 (0.65 – 0.80) | 0.65 (0.58 – 0.72) |  |  |  |  |
| 16:0 | CAM | 13.7 (12.4 – 15.1) | 14.3 (13.0 – 15.7) | 14.4 (13.0 – 15.7) |  | 0.232 | 0.392 | 0.121 |
|  | OLA | 14.8 (13.5 – 16.1) | 14.2 (12.9 – 15.6) | 14.3 (13.0 – 15.7) |  |  |  |  |
|  | FLX | 15.2 (13.8 – 16.5) | 17.0 (15.6 – 18.3) | 13.5 (12.2 – 14.9) |  |  |  |  |
|  | Mean | 14.6 (13.8 – 15.3) | 15.2 (14.4 – 16.0) | 14.1 (13.3 – 14.9) |  |  |  |  |
| 18:0 | CAM | 14.2 (13.2 – 15.2) | 13.2 (12.3 – 14.2) | 14.4 (13.4 – 15.4) |  | 0.003 | 0.399 | 0.547 |
|  | OLA | 15.7 (14.7 – 16.9) | 14.3 (13.4 – 15.4) | 15.0 (14.0 – 16.1) |  |  |  |  |
|  | FLX | 15.9 (14.8 – 17.1) | 13.9 (13.0 – 14.9) | 14.3 (13.3 – 15.3) |  |  |  |  |
|  | Mean | 15.3^a^ (14.6 – 15.9) | 13.8^b^ (13.3 – 14.4) | 14.6^ab^ (14.0 – 15.2) |  |  |  |  |
| 20:0 | CAM | 3.08 (2.37 – 3.78) | 2.76 (2.05 – 3.46) | 4.22 (3.52 – 4.93) |  | 0.003 | 0.514 | 0.688 |
|  | OLA | 3.62 (2.92 – 4.33) | 3.01 (2.30 – 3.71) | 3.80 (3.10 – 4.51) |  |  |  |  |
|  | FLX | 3.50 (2.79 – 4.20) | 2.86 (2.16 – 3.57) | 4.39 (3.68 – 5.09) |  |  |  |  |
|  | Mean | 3.40^b^ (2.99 – 3.80) | 2.88^b^ (2.47 – 3.28) | 4.14^a^ (3.73 – 4.54) |  |  |  |  |
| 21:0 | CAM | 0.26 (0.11 – 0.59) | 0.38 (0.23 – 0.65) | 0.29 (0.17 – 0.49) |  | 0.617 | 0.256 | 0.587 |
|  | OLA | 0.30 (0.15 – 0.60) | 0.29 (0.14 – 0.60) | 0.27 (0.15 – 0.48) |  |  |  |  |
|  | FLX | 0.22 (0.11 – 0.45) | 0.24 (0.14 – 0.42) | 0.49 (0.27 – 0.92) |  |  |  |  |
|  | Mean | 0.26 (0.17 – 0.40) | 0.30 (0.21 – 0.43) | 0.34 (0.24 – 0.47) |  |  |  |  |
| 22:0 | CAM | 2.95 (2.39 – 3.63) | 3.26 (2.65 – 4.02) | 3.78 (3.07 – 4.66) |  | 0.009 | 0.497 | 0.757 |
|  | OLA | 2.71 (2.20 – 3.34) | 3.66 (2.97 – 4.51) | 3.70 (3.00 – 4.55) |  |  |  |  |
|  | FLX | 3.29 (2.67 – 4.06) | 3.35 (2.72 – 4.13) | 4.20 (3.41 – 5.17) |  |  |  |  |
|  | Mean | 2.97^b^ (2.64 – 3.36) | 3.42^ab^ (3.03 – 3.86) | 3.89^a^ (3.45 – 4.38) |  |  |  |  |
| 23:0 | CAM | 0.66 (0.54 – 0.81) | 1.13 (0.92 – 1.39) | 0.74 (0.60 – 0.91) |  | <0.001 | 0.620 | 0.351 |
|  | OLA | 0.86 (0.70 – 1.06) | 1.21 (0.99 – 1.49) | 0.70 (0.57 – 0.87) |  |  |  |  |
|  | FLX | 0.71 (0.58 – 0.87) | 0.97 (0.79 – 1.19) | 0.79 (0.63 – 0.98) |  |  |  |  |
|  | Mean | 0.74^b^ (0.66 – 0.83) | 1.10^a^ (0.98 – 1.24) | 0.74^b^ (0.66 – 0.84) |  |  |  |  |
| 24:0 | CAM | 3.71 (2.06 – 5.36) | 5.81 (4.16 – 7.46) | 4.64 (2.99 – 6.30) |  | 0.041 | 0.909 | 0.986 |
|  | OLA | 3.72 (2.07 – 5.37) | 6.05 (4.40 – 7.70) | 5.07 (3.42 – 6.72) |  |  |  |  |
|  | FLX | 4.01 (2.36 – 5.66) | 5.37 (3.72 – 7.02) | 4.94 (3.29 – 6.59) |  |  |  |  |
|  | Mean | 3.81^b^ (2.86 – 4.77) | 5.74^a^ (4.79 – 6.69) | 4.88^ab^ (3.93 – 5.84) |  |  |  |  |
| $\sum$ SFA | CAM | 39.8 (36.5 – 43.1) | 42.7 (39.4 – 46.0) | 43.9 (40.6 – 47.2) |  | 0.366 | 0.954 | 0.842 |
|  | OLA | 43.1 (39.8 – 46.4) | 44.2 (40.9 – 47.5) | 44.3 (41.0 – 47.6) |  |  |  |  |
|  | FLX | 44.3 (41.0 – 47.6) | 45.7 (42.4 – 49.0) | 44.5 (41.2 – 47.8) |  |  |  |  |
|  | Mean | 42.4 (40.5 – 44.3) | 44.2 (42.3 – 46.1) | 44.2 (42.3 – 46.1) |  |  |  |  |
| 16:1c9 | CAM | 2.33 (1.80 – 2.85) | 2.24 (1.72 – 2.77) | 2.43 (1.91 – 2.96) |  | 0.703 | 0.833 | 0.668 |
|  | OLA | 2.21 (1.69 – 2.74) | 2.00 (1.48 – 2.53) | 2.34 (1.81 – 2.86) |  |  |  |  |
|  | FLX | 2.22 (1.70 – 2.75) | 2.16 (1.63 – 2.68) | 1.97 (1.44 – 2.49) |  |  |  |  |
|  | Mean | 2.26 (1.95 – 2.56) | 2.13 (1.83 – 2.44) | 2.25 (1.94 – 2.55) |  |  |  |  |
| 18:1n9 | CAM | 12.3 (10.9 – 13.9) | 13.0 (11.5 – 14.6) | 12.0 (10.7 – 13.5) |  | 0.133 | 0.208 | 0.379 |
|  | OLA | 12.1 (10.7 – 13.6) | 12.6 (11.2 – 14.2) | 13.4 (11.9 – 15.1) |  |  |  |  |
|  | FLX | 11.5 (10.2 – 13.0) | 13.7 (12.2 – 15.5) | 12.2 (10.8 – 13.7) |  |  |  |  |
|  | Mean | 12.0 (11.2 – 12.8) | 13.1 (12.2 – 14.0) | 12.5 (11.7 – 13.4) |  |  |  |  |
| 18:1c11 | CAM | 2.26 (1.92 – 2.66) | 1.98 (1.69 – 2.34) | 2.05 (1.74 – 2.42) |  | 0.754 | 0.158 | 0.765 |
|  | OLA | 2.27 (1.93 – 2.67) | 2.15 (1.82 – 2.53) | 2.37 (2.01 – 2.79) |  |  |  |  |
|  | FLX | 2.16 (1.83 – 2.54) | 2.22 (1.89 – 2.62) | 2.18 (1.85 – 2.56) |  |  |  |  |
|  | Mean | 2.23 (2.03 – 2.45) | 2.12 (1.93 – 2.33) | 2.20 (2.00 – 2.41) |  |  |  |  |
| 20:1c11 | CAM | 1.78 (1.59 – 1.99) | 1.97 (1.76 – 2.20) | 1.99 (1.78 – 2.23) |  | 0.090 | 0.874 | 0.358 |
|  | OLA | 1.95 (1.74 – 2.18) | 1.76 (1.57 – 1.97) | 1.96 (1.75 – 2.20) |  |  |  |  |
|  | FLX | 1.78 (1.59 – 2.00) | 1.55 (1.39 – 1.74) | 1.93 (1.72 – 2.16) |  |  |  |  |
|  | Mean | 1.83 (1.72 – 1.96) | 1.75 (1.64 – 1.87) | 1.96 (1.84 – 2.09) |  |  |  |  |
| 24:1n9 | CAM | 1.60 (1.34 – 1.85) | 1.69 (1.44 – 1.95) | 1.69 (1.44 – 1.95) |  | 0.425 | 0.893 | 0.976 |
|  | OLA | 1.59 (1.33 – 1.84) | 1.64 (1.39 – 1.90) | 1.69 (1.44 – 1.95) |  |  |  |  |
|  | FLX | 1.55 (1.30 – 1.81) | 1.61 (1.36 – 1.87) | 1.77 (1.51 – 2.02) |  |  |  |  |
|  | Mean | 1.58 (1.43 – 1.73) | 1.65 (1.50 – 1.79) | 1.72 (1.57 – 1.86) |  |  |  |  |
| $\sum$ MUFA | CAM | 20.3 (18.3 – 22.4) | 20.8 (18.8 – 23.1) | 20.2 (18.2 – 22.3) |  | 0.282 | 0.285 | 0.445 |
|  | OLA | 20.1 (18.2 – 22.3) | 20.3 (18.4 – 22.5) | 21.8 (19.7 – 24.1) |  |  |  |  |
|  | FLX | 19.2 (17.4 – 21.3) | 21.5 (19.4 – 23.8) | 20.1 (18.2 – 22.2) |  |  |  |  |
|  | Mean | 19.9 (18.7 – 21.1) | 20.9 (19.7 – 22.1) | 20.7 (19.5 – 21.9) |  |  |  |  |
| 18:2n6 | CAM | 23.6 (21.5 – 25.8) | 20.3 (18.1 – 22.5) | 20.4 (18.2 – 22.6) |  | 0.001 | 0.332 | 0.900 |
|  | OLA | 21.6 (19.4 – 23.8) | 17.5 (15.3 – 19.7) | 19.6 (17.5 – 21.8) |  |  |  |  |
|  | FLX | 20.8 (18.6 – 23.0) | 16.6 (14.5 – 18.8) | 18.5 (16.3 – 20.7) |  |  |  |  |
|  | Mean | 22.0^a^ (20.8 – 23.3) | 18.2^b^ (16.9 – 19.4) | 19.5^b^ (18.3 – 20.8) |  |  |  |  |
| 18:3n3 | CAM | 0.69 (0.47 – 0.90) | 0.95 (0.74 – 1.17) | 0.97 (0.75 – 1.18) |  | 0.015 | 0.084 | 0.079 |
|  | OLA | 0.66 (0.44 – 0.87) | 1.07 (0.85 – 1.28) | 0.71 (0.50 – 0.93) |  |  |  |  |
|  | FLX | 0.63 (0.42 – 0.85) | 0.65 (0.43 – 0.87) | 0.91 (0.69 – 1.13) |  |  |  |  |
|  | Mean | 0.66^b^ (0.54 – 0.78) | 0.89^a^ (0.77 – 1.01) | 0.86^a^ (0.74 – 0.99) |  |  |  |  |
| 18:3n6 | CAM | 0.42 (0.28 – 0.62) | 0.50 (0.34 – 0.73) | 0.53 (0.36 – 0.79) |  | 0.360 | 0.283 | 0.449 |
|  | OLA | 0.52 (0.35 – 0.77) | 0.75 (0.51 – 1.11) | 0.38 (0.26 – 0.57) |  |  |  |  |
|  | FLX | 0.45 (0.31 – 0.67) | 0.46 (0.31 – 0.69) | 0.38 (0.24 – 0.60) |  |  |  |  |
|  | Mean | 0.46 (0.37 – 0.58) | 0.56 (0.44 – 0.70) | 0.43 (0.34 – 0.54) |  |  |  |  |
| 18:4n3 | CAM | 0.41 (0.01 – 20.5) | 0.81 (0.12 – 5.34) | 0.11 (0.06 – 0.21) |  | 0.379 | 0.091 | 0.552 |
|  | OLA | 0.29 (0.08 – 1.11) | 0.07 (0.02 – 0.26) | 0.06 (0.03 – 0.11) |  |  |  |  |
|  | FLX | 0.11 (0.05 – 0.24) | 0.13 (0.02 – 0.73) | 0.07 (0.03 – 0.15) |  |  |  |  |
|  | Mean | 0.23 (0.09 – 0.62) | 0.19 (0.07 – 0.49) | 0.08 (0.05 – 0.11) |  |  |  |  |
| 20:2n6 | CAM | 1.02 (0.80 – 1.30) | 1.03 (0.81 – 1.32) | 1.06 (0.83 – 1.36) |  | 0.721 | 0.256 | 0.300 |
|  | OLA | 0.94 (0.73 – 1.19) | 1.01 (0.79 – 1.29) | 0.84 (0.66 – 1.07) |  |  |  |  |
|  | FLX | 0.87 (0.68 – 1.12) | 0.86 (0.67 – 1.10) | 1.15 (0.90 – 1.47) |  |  |  |  |
|  | Mean | 0.94 (0.82 – 1.08) | 0.97 (0.84 – 1.11) | 1.01 (0.88 – 1.16) |  |  |  |  |
| 20:3n9 | CAM | 2.65 (2.00 – 2.51) | 2.74 (2.07 – 3.64) | 2.60 (1.93 – 3.50) |  | 0.625 | 0.712 | 0.459 |
|  | OLA | 2.26 (1.68 – 3.04) | 2.73 (2.06 – 3.62) | 2.13 (1.61 – 2.82) |  |  |  |  |
|  | FLX | 2.46 (1.85 – 3.26) | 2.62 (1.97 – 3.47) | 3.10 (2.31 – 4.18) |  |  |  |  |
|  | Mean | 2.45 (2.08 – 2.89) | 2.70 (2.29 – 3.17) | 2.58 (2.18 – 3.06) |  |  |  |  |
| 20:3n6 | CAM | 3.32 (2.92 – 3.77) | 2.73 (2.41 – 3.10) | 2.93 (2.58 – 3.33) |  | 0.002 | 0.572 | 0.025 |
|  | OLA | 3.11 (2.74 – 3.53) | 3.10 (2.73 – 3.53) | 2.97 (2.61 – 3.37) |  |  |  |  |
|  | FLX | 3.28^a^ (2.88 – 3.72) | 2.46^b^ (2.17 – 2.79) | 3.18^a^ (2.80 – 3.62) |  |  |  |  |
|  | Mean | 3.23^a^ (3.00 – 3.48) | 2.75^b^ (2.56 – 2.96) | 3.03^ab^ (2.81 – 3.26) |  |  |  |  |
| 20:4n6 | CAM | 3.90 (3.41 – 4.46) | 3.29 (2.88 – 3.77) | 3.15 (2.76 – 3.60) |  | 0.007 | 0.770 | 0.610 |
|  | OLA | 3.57 (3.12 – 4.08) | 3.23 (2.82 – 3.69) | 3.37 (2.95 – 3.85) |  |  |  |  |
|  | FLX | 3.65 (3.19 – 4.17) | 3.39 (2.97 – 3.88) | 3.14 (2.74 – 3.58) |  |  |  |  |
|  | Mean | 3.70^a^ (3.43 – 4.00) | 3.30^b^ (3.06 – 3.57) | 3.22^b^ (2.98 – 3.47) |  |  |  |  |
| 20:3n3 | CAM | 0.21 (0.17 – 0.26) | 0.22 (0.18 – 0.27) | 0.30 (0.25 – 0.37) |  | 0.023 | 0.065 | 0.431 |
|  | OLA | 0.21 (0.16 – 0.26) | 0.22 (0.18 – 0.28) | 0.23 (0.19 – 0.28) |  |  |  |  |
|  | FLX | 0.20 (0.16 – 0.24) | 0.24 (0.20 – 0.30) | 0.27 (0.21 – 0.34) |  |  |  |  |
|  | Mean | 0.21^b^ (0.18 – 0.23) | 0.23^ab^ (0.20 – 0.26) | 0.26^a^ (0.23 – 0.30) |  |  |  |  |
| 20:5n3 | CAM | 1.21 (0.86 – 1.69) | 1.34 (0.96 – 1.88) | 1.45^xy^ (1.03 – 2.03) |  | 0.268 | 0.049 | 0.332 |
|  | OLA | 1.12 (0.80 – 1.57) | 1.57 (1.12 – 2.20) | 1.10^y^ (0.78 – 1.54) |  |  |  |  |
|  | FLX | 1.28 (0.91 – 1.80) | 1.38 (0.99 – 1.94) | 2.09^x^ (1.49 – 2.93) |  |  |  |  |
|  | Mean | 1.20 (0.99 – 1.46) | 1.43 (1.17 – 1.73) | 1.49 (1.23 – 1.81) |  |  |  |  |
| 22:2n6 | CAM | 0.57 (0.43 – 0.75) | 0.55 (0.42 – 0.73) | 0.85 (0.65 – 1.12) |  | 0.023 | 0.301 | 0.403 |
|  | OLA | 0.53 (0.40 – 0.70) | 0.65 (0.50 – 0.85) | 0.62 (0.47 – 0.82) |  |  |  |  |
|  | FLX | 0.57 (0.43 – 0.74) | 0.62 (0.48 – 0.82) | 0.81 (0.61 – 1.06) |  |  |  |  |
|  | Mean | 0.55^b^ (0.47 – 0.65) | 0.61^ab^ (0.52 – 0.71) | 0.75^a^ (0.64 – 0.88) |  |  |  |  |
| 22:4n6 | CAM | 0.19 (0.16 – 0.23) | 0.15 (0.12 – 0.20) | 0.19 (0.16 – 0.23) |  | 0.179 | 0.197 | 0.096 |
|  | OLA | 0.20 (0.16 – 0.26) | 0.21 (0.17 – 0.27) | 0.16 (0.13 – 0.19) |  |  |  |  |
|  | FLX | 0.19 (0.16 – 0.23) | 0.14 (0.11 – 0.18) | 0.18 (0.14 – 0.22) |  |  |  |  |
|  | Mean | 0.20 (0.17 – 0.22) | 0.17 (0.15 – 0.19) | 0.17 (0.16 – 0.19) |  |  |  |  |
| 22:3n3 | CAM | 0.35 (0.19 – 0.63) | 0.98 (0.56 – 1.70) | 0.48 (0.21 – 1.06) |  | 0.001 | 0.428 | 0.172 |
|  | OLA | 0.66 (0.36 – 1.19) | 1.24 (0.64 – 2.38) | 0.18 (0.10 – 0.33) |  |  |  |  |
|  | FLX | 0.51 (0.27 – 0.98) | 0.88 (0.48 – 1.60) | 0.35 (0.14 – 0.89) |  |  |  |  |
|  | Mean | 0.49^b^ (0.34 – 0.70) | 1.02^a^ (0.72 – 1.45) | 0.31^b^ (0.20 – 0.49) |  |  |  |  |
| 22:5n6 | CAM | 0.11 (0.06 – 0.16) | 0.00 (0.00 – 0.05) | 0.07 (0.02 – 0.11) |  | 0.520 | 0.908 | 0.151 |
|  | OLA | 0.02 (0.00 – 0.06) | 0.04 (0.00 – 0.09) | 0.05 (0.00 – 0.10) |  |  |  |  |
|  | FLX | 0.03 (0.00 – 0.07) | 0.04 (0.00 – 0.08) | 0.04 (0.00 – 0.09) |  |  |  |  |
|  | Mean | 0.05 (0.03 – 0.08) | 0.03 (0.00 – 0.05) | 0.05 (0.03 – 0.08) |  |  |  |  |
| 22:5n3 | CAM | 0.83 (0.70 – 0.97) | 0.66 (0.56 – 0.78) | 0.77 (0.66 – 0.91) |  | 0.001 | 0.265 | 0.500 |
|  | OLA | 0.81 (0.69 – 0.95) | 0.71 (0.60 – 0.85) | 0.84 (0.71 – 0.99) |  |  |  |  |
|  | FLX | 0.86 (0.73 – 1.02) | 0.59 (0.50 – 0.69) | 0.75 (0.63 – 0.89) |  |  |  |  |
|  | Mean | 0.83^a^ (0.76 – 0.91) | 0.65^b^ (0.59 – 0.72) | 0.79^a^ (0.71 – 0.86) |  |  |  |  |
| 22:6n3 | CAM | 0.20 (0.14 – 0.30) | 0.37 (0.23 – 0.59) | 0.14 (0.10 – 0.21) |  | 0.006 | 0.092 | 0.046 |
|  | OLA | 0.42 (0.26 – 0.67) | 0.33 (0.21 – 0.50) | 0.17 (0.12 – 0.25) |  |  |  |  |
|  | FLX | 0.15 (0.08 – 0.28) | 0.39 (0.27 – 0.58) | 0.26 (0.16 – 0.42) |  |  |  |  |
|  | Mean | 0.23^ab^ (0.17 – 0.31) | 0.36^a^ (0.28 – 0.46) | 0.18^b^ (0.15 – 0.23) |  |  |  |  |
| $\sum$ PUFA | CAM | 39.8 (37.4 – 42.2) | 36.3 (33.9 – 38.7) | 35.8 (33.4 – 38.2) |  | 0.010 | 0.284 | 0.498 |
|  | OLA | 36.7 (34.3 – 39.1) | 35.3 (32.9 – 37.7) | 33.8 (31.4 – 36.2) |  |  |  |  |
|  | FLX | 36.4 (34.0 – 38.8) | 32.2 (29.8 – 34.5) | 35.3 (32.9 – 37.6) |  |  |  |  |
|  | Mean | 37.6^a^ (36.3 – 39.0) | 34.6^b^ (33.2 – 36.0) | 35.0^b^ (33.6 – 36.3) |  |  |  |  |
| $\sum$ n-6 | CAM | 33.4 (31.1 – 35.7) | 28.8 (26.5 – 31.1) | 29.3 (27.0 – 31.7) |  | <0.001 | 0.476 | 0.868 |
|  | OLA | 30.8 (28.5 – 33.1) | 26.7 (24.4 – 29.0) | 28.2 (25.8 – 30.5) |  |  |  |  |
|  | FLX | 30.1 (27.8 – 32.4) | 24.9 (22.6 – 27.2) | 27.8 (25.5 – 30.1) |  |  |  |  |
|  | Mean | 31.4^a^ (30.1 – 32.8) | 26.8^b^ (25.5 – 28.1) | 28.4^b^ (27.1 – 29.8) |  |  |  |  |
| $\sum$ n-3 | CAM | 3.63 (3.12 – 4.23) | 4.65 (4.00 – 5.42) | 4.07 (3.49 – 4.73) |  | <0.001 | 0.051 | 0.031 |
|  | OLA | 3.73^ab^ (3.20 – 4.34) | 5.05^a^ (4.33 – 5.88) | 3.40^b^ (2.92 – 3.96) |  |  |  |  |
|  | FLX | 3.60 (3.09 – 4.19) | 4.31 (3.70 – 5.01) | 4.57 (3.93 – 5.32) |  |  |  |  |
|  | Mean | 3.65^b^ (3.34 – 3.99) | 4.66^a^ (4.27 – 5.09) | 3.99^b^ (3.65 – 4.35) |  |  |  |  |
| $\sum$ n-6/n-3 | CAM | 9.29 (8.11 – 10.5) | 6.22 (5.04 – 7.39) | 7.34 (6.16 – 8.51) |  | <0.001 | 0.062 | 0.248 |
|  | OLA | 8.46 (7.28 – 9.64) | 5.60 (4.42 – 6.78) | 8.33 (7.16 – 9.51) |  |  |  |  |
|  | FLX | 8.51 (7.34 – 9.69) | 5.85 (4.67 – 7.03) | 6.35 (5.18 – 7.53) |  |  |  |  |
|  | Mean | 8.75^a^ (8.07 – 9.43) | 5.89^c^ (5.21 – 6.57) | 7.34^b^ (6.66 – 8.02) |  |  |  |  |

Abbreviations: SFA = saturated fatty acids; MUFA = monounsaturated fatty acids; PUFA = polyunsaturated fatty acids; Trmt = treatment

^1^Mean indicates week data pooled across treatments

^2^Treatment effects were obtained only from the week 16 data

^a,b,c^Values in a row with different superscripts differ across time for a particular treatment group (*P* < 0.05)

^xy^Values in a column with different superscripts differ across treatments for a particular fatty acid at week 16 (P < 0.05)
